# Estimated Incidence Rate of Specific Types of Cardiovascular and Respiratory Hospitalizations Attributable to Respiratory Syncytial Virus Among Adults in Germany Between 2015 and 2019

**DOI:** 10.1111/irv.70097

**Published:** 2025-04-28

**Authors:** Caihua Liang, Aleksandra Polkowska‐Kramek, Caroline Lade, Lea J. Bayer, Robin Bruyndonckx, Bennet Huebbe, Worku Biyadgie Ewnetu, Pimnara Peerawaranun, Maribel Casas, Thao Mai Phuong Tran, Gordon Brestrich, Christof von Eiff, Bradford D. Gessner, Elizabeth Begier, Gernot Rohde

**Affiliations:** ^1^ Pfizer Inc New York New York USA; ^2^ P95 Epidemiology & Pharmacovigilance Leuven Belgium; ^3^ Pfizer Pharma GmbH Berlin Germany; ^4^ Pfizer Inc Dublin Ireland; ^5^ University Hospital, Medical Clinic I, Department of Respiratory Medicine Goethe University Frankfurt Frankfurt Germany

**Keywords:** cardiorespiratory, Germany, hospitalization, quasi‐Poisson, RSV, time‐series modeling

## Abstract

**Background:**

RSV incidence in adults is frequently underestimated due to non‐specific symptomatology, limited standard‐of‐care testing, and lower test sensitivity compared to infants. We conducted a retrospective observational study to estimate RSV‐attributable incidence of specific types of cardiorespiratory hospitalizations among adults in Germany between 2015 and 2019.

**Methods:**

Information on hospitalizations and the number of people at risk of hospitalization (denominator) was gathered from a Statutory Health Insurance database. A quasi‐Poisson regression model accounting for periodic and aperiodic time trends and virus activity was fitted to estimate the RSV‐attributable incidence rate (IR) of four specific cardiovascular hospitalizations (arrhythmia, ischemic heart diseases, chronic heart failure exacerbations, and cerebrovascular diseases) and four specific respiratory hospitalizations (influenza/pneumonia, bronchitis/bronchiolitis, chronic lower respiratory tract diseases, and upper respiratory tract diseases).

**Results:**

The estimated RSV‐attributable IRs of hospitalizations generally increased with age. Among estimated cardiovascular hospitalizations in adults aged ≥ 60 years, arrhythmia and ischemic heart diseases accounted for the highest incidence of RSV‐attributable events, followed by chronic heart failure exacerbation, with annual IR ranges of 157–260, 133–214, and 105–169 per 100,000 person‐years, respectively. The most frequent RSV‐attributable respiratory hospitalizations in adults aged ≥ 60 years were estimated for chronic lower respiratory tract diseases and bronchitis/bronchiolitis, with annual IR ranges of 103–168 and 77–122 per 100,000 person‐years, respectively.

**Conclusions:**

RSV causes a considerable burden of respiratory and cardiovascular hospitalizations in adults in Germany, similar to other respiratory viruses (e.g., influenza and SARS‐CoV‐2). This highlights the need to implement effective prevention strategies, especially for older adults.

## 1 Introduction

The incidence rate (IR) and clinical burden of RSV disease in adults are challenging to measure due to symptoms resembling influenza and other co‐circulating respiratory viruses [1]. Limited standard‐of‐care RSV testing among adults, single specimen collection (i.e., use of nasopharyngeal/nasal swab only), and reduced sensitivity of diagnostic testing compared to children [2, 3, 4, 5] contribute to the underestimation of the RSV burden. Additionally, inadequate RSV diagnostic capacity in many healthcare facilities [1, 4, 6, 7] and low awareness of RSV, particularly for patients with cardiopulmonary manifestations, hinder accurate RSV burden measurement [8]. Further, even when RSV is diagnosed via laboratory testing, other non‐specific acute respiratory infection codes may be used for administrative diagnoses [9].

RSV‐associated exacerbation of underlying cardiopulmonary diseases may contribute to a large and unrecognized burden of RSV disease in adults [8]. Several studies have demonstrated that RSV can cause exacerbations of asthma and chronic obstructive pulmonary disease (COPD) [10, 11, 12, 13, 14]. Growing evidence indicates that RSV also is associated with cardiovascular diseases such as acute myocardial infarction, stroke, arrhythmia, exacerbations of congestive heart failure (CHF), and coronary artery disease (CAD) [8, 15, 16, 17, 18, 19, 20, 21, 22]. Nonetheless, estimates of the burden of RSV‐attributable cardiovascular hospitalizations in adults are limited.

In Germany, data on the RSV burden from prospective observational incidence studies are unavailable [23], but a recent model‐based study of cardiorespiratory hospitalizations and deaths showed considerable RSV morbidity and mortality between 2015 and 2019 [24]. However, detailed data on RSV‐attributable hospitalization incidence for more specific respiratory and cardiovascular subgroups remain important gaps in knowledge, impeding health economic evaluations and efficient vaccine policies. We conducted a model‐based study to estimate RSV‐attributable hospitalizations for four respiratory (influenza/pneumonia, bronchitis/bronchiolitis, chronic lower respiratory tract diseases, and upper respiratory tract diseases) and four cardiovascular (chronic heart failure exacerbation, ischemic heart diseases, arrhythmia, and cerebrovascular diseases) conditions in Germany.

## 2 Methods

### 2.1 Study Design

We conducted an observational retrospective and modeling study to estimate the IRs of specific respiratory and cardiovascular hospitalizations attributable to RSV among adults in Germany using a quasi‐Poisson regression model.

### 2.2 Data Sources

Data on hospitalizations were sourced from the Deutsche Analysedatenbank für Evaluation und Versorgungsforschung (DADB) database from Gesundheitsforen Leipzig (GFL). This database contains Statutory Health Insurance (SHI) data of approximately 3.5 million insured individuals. Participants included in the study were adults aged ≥ 18 years. The age and sex structure of the DADB database is similar to that of the German population, with adjustment factors to account for a slightly younger population in DADB available [25]. The study period, from 2015 to 2019, was selected to avoid any distortions in RSV epidemiology due to the COVID‐19 pandemic [26].

The study included four specific respiratory outcomes (influenza/pneumonia [J10–J18], bronchitis/bronchiolitis [J20–J22], chronic lower respiratory tract diseases [J40–J47], and upper respiratory tract diseases [J00–J06, J30–J39]) and four specific cardiovascular outcomes (chronic heart failure [I42–I43, I50, I51.7], ischemic heart diseases [I20–I25], arrhythmias [I44–I49], and cerebrovascular diseases [I60–I68]). To compare the difference between the number of observed (reported in the database) and attributable (model‐based) RSV events, we obtained RSV‐specific hospitalizations (B97.4, J21.0, J12.1, J20.5) stratified by age group.

Hospitalization was defined as an overnight stay in a hospital commencing from the admission date. If subsequent hospitalizations for the same outcome occurred within 30 days from discharge, these were collapsed with the initial hospitalization to avoid overcounting cases due to readmission.

Individuals were categorized into four age groups: 18–44, 45–59, 60–74, and ≥ 75 years. For anonymization purposes, data were suppressed if fewer than 5 cases were reported in the age group and outcome stratum.

The indicator for RSV circulation was defined as the weekly number of RSV‐related hospitalizations in children under 2 years of age (ICD‐10‐GM codes: B97.4, J21.0, J12.1, J20.5, J21.9). As in other studies [6, 22, 27, 28], we selected RSV circulation in children due to the higher frequency of testing and hospitalization in this age group and the higher sensitivity of diagnostic tests compared to adults [4, 29]. As most bronchiolitis cases in young children are caused by RSV, we also included J21.9 (acute bronchiolitis, unspecified) [30, 31, 32] as a proxy for RSV circulation. The indicator for influenza circulation was defined as the weekly number of influenza‐specific hospitalizations (ICD‐10‐GM codes: J09, J10, J11) in adults aged ≥ 60 years [28].

### 2.3 Statistical Analysis

Upon observation of seasonality, outcome‐specific age‐group‐stratified weekly aggregated data were modeled using a quasi‐Poisson regression model. The model accounts for baseline periodic and aperiodic time trends, viral activity (RSV and influenza), and potential overdispersion. The final model for each outcome and age group combination was constructed in a step‐by‐step manner, as described in the previously published protocol [33]. In summary, a model containing periodic and aperiodic trends was fitted to each outcome‐specific age‐stratified dataset, after which the polynomial order was reduced when possible (significance level 0.05) and viral indicators were added one by one (until each viral indicator was included).

With these fitted models, we obtained the annual number of RSV‐attributable events and the proportion of RSV‐attributable events for each outcome and age group as described in the protocol [33]. The results for the age group ≥ 60 years were based on pooling results for models fitted to adults for included age groups (60–74 years, ≥ 75 years); thus, confidence intervals are not provided.

Yearly IRs of RSV‐attributable events, expressed in the number of events per 100,000 person‐years, were obtained by dividing the annual model‐based number of RSV‐attributable events by the population at risk. This yearly age‐specific population at risk of the event was obtained from the DADB database. Because the DADB database, from which our estimates were obtained, has a slightly younger population compared to the overall German population covered by SHI, the yearly RSV‐attributable events were multiplied by age‐specific correction factors based on data provided by GFL to scale the IRs to a nationally representative level.

Confidence intervals were obtained via residual bootstrapping. All data management and statistical analyses were conducted using R software (version 4.0.4).

### 2.4 Ethical Considerations

This study used aggregated and anonymized data; therefore, it did not require approval from institutional review boards or ethical committees or informed consent from patients. The study was conducted following legal and regulatory requirements and research practices described in the Good Epidemiological Practice guidelines issued by the International Epidemiological Association [34].

## 3 Results

### 3.1 Reported Hospitalizations

Between 2015 and 2019, the largest number of reported hospitalizations was due to arrhythmia and ischemic heart diseases, responsible for 266,463 and 259,052 hospitalizations, respectively. Chronic lower respiratory tract diseases were responsible for 162,344 hospitalizations, with influenza/pneumonia (74,064 hospitalizations) accounting for the most (Table S1). For different subgroups, 70% or more of hospitalizations involved individuals aged ≥ 60 years. The exception to this trend was observed for upper respiratory tract diseases, where most hospitalizations (74% or more) occurred in adults aged 18–59 years.

The unsuppressed reported number of RSV‐specific hospitalizations based on ICD code diagnoses alone throughout the study period was 24 cases combined for all age groups. However, the total number of RSV‐specific hospitalizations is unknown since, during most weeks in which RSV‐specific hospitalizations were reported, counts were below five and, hence, suppressed for anonymity.

### 3.2 Estimated RSV‐Attributable Hospitalizations

The estimated IR of RSV‐attributable diseases fluctuated year to year, with the highest incidence observed for most outcomes in 2017. Due to a lack of adequate seasonal fluctuations, several outcomes were not modeled in the youngest age group or yielded broad confidence intervals. Additionally, small point estimates were associated with confidence intervals including zero. For cerebrovascular diseases, only data for the age group ≥ 75 years were suitable for modeling (Tables 1 and 2).

**TABLE 1:** Estimated incidence rate per 100,000 person‐years of RSV‐attributable respiratory hospitalizations and percentage (%) of all respective respiratory hospitalizations attributable to RSV infections in adults in Germany, 2015–2019.

Age groups (years)	2015		2016		2017		2018		2019	
	IR [95% CI]	%	IR [95% CI]	%	IR [95% CI]	%	IR [95% CI]	%	IR [95% CI]	%
Influenza/pneumonia										
18–44	2.7 [0, 6.0]	2.9	4.2 [0, 9.1]	3.6	4.8 [0, 10.4]	4.4	3.8 [0, 8.4]	3.1	5.0 [0, 11.0]	4.6
45–59	3.3 [0, 8.1]	1.3	4.8 [0, 11.8]	1.8	5.2 [0, 13.0]	1.9	4.1 [0, 10.3]	1.4	5.5 [0, 13.6]	2.0
60–74	20.4 [0, 42.6]	1.9	20.8 [0, 43.6]	1.8	27.7 [0, 57.9]	2.4	18.1 [0, 37.9]	1.6	23.9 [0, 50.0]	2.2
≥ 75	231.3 [137.4, 330.0]	4.3	253.7 [150.7, 362.0]	4.5	292.4 [173.7, 417.2]	5.1	205.8 [122.3, 293.7]	3.6	273.9 [162.7, 390.8]	5.0
≥ 60^a^	73.7	3.4	81.0	3.5	97.9	4.1	67	2.8	88.4	3.9
Bronchitis/bronchiolitis										
18–44	0.4 [0, 2.7]	0.9	0.5 [0, 3.3]	1.0	0.6 [0, 4.5]	1.4	0.4 [0, 3.2]	0.9	0.6 [0, 4.3]	1.5
45–59	6.6 [3.4, 10.1]	8.6	7.7 [4.0, 11.9]	9.2	10.0 [5.2, 15.4]	11.0	6.9 [3.6, 10.7]	7.8	9.7 [5.0, 14.9]	11.1
60–74	25.5 [14.9, 35.8]	9.0	24.1 [14.1, 33.9]	8.8	34.4 [20.1, 48.4]	11.8	21.2 [12.4, 29.8]	7.3	28.5 [16.7, 40.1]	10.9
≥ 75	283.6 [222.9, 343.8]	19.6	281.9 [221.6, 341.8]	20.1	359.8 [282.8, 436.3]	22.4	234.4 [184.2, 284.2]	16.6	314.2 [247.0, 381.0]	24.5
≥ 60^a^	91.3	15.5	91.5	15.9	121.8	18.7	77.2	13.1	102.9	19.3
Chronic lower respiratory tract diseases										
18–44	NA		NA		NA		NA		NA	
45–59	18.9 [1.7, 35.5]	2.4	17.4 [1.5, 32.7]	2.0	28.1 [2.5, 52.8]	3.1	17.5 [1.6, 32.9]	2.0	25.4 [2.3, 47.8]	2.9
60–74	74.4 [17.2, 133.8]	2.4	70.4 [16.3, 126.6]	2.1	100.5 [23.2, 180.7]	3.0	61.8 [14.3, 111.2]	1.9	83.3 [19.2, 149.8]	2.6
≥ 75	279.5 [125.7, 432.5]	3.3	239.0 [107.5, 369.8]	2.6	342.7 [154.1, 530.3]	3.9	211.4 [95.1, 327.1]	2.6	293.0 [131.7, 453.3]	3.6
≥ 60^a^	128.9	2.8	116.7	2.3	168.2	3.4	102.9	2.2	140.1	3.1
Upper respiratory tract diseases										
18–44	10.9 [0, 27.3]	1.7	10.4 [0, 26.1]	1.5	17.4 [0, 43.7]	2.4	11.1 [0, 27.8]	1.6	15.8 [0, 39.7]	2.3
45–59	8.2 [0, 21.7]	1.9	8.1 [0, 21.3]	1.7	12.4 [0, 32.9]	2.5	8.0 [0, 21.1]	1.7	11.4 [0, 30.1]	2.4
60–74	18.6 [0.4, 35.5]	3.6	17.6 [0.4, 33.6]	3.1	25.1 [0.6, 48.0]	4.4	15.4 [0.4, 29.5]	2.8	20.8 [0.5, 39.8]	3.9
≥ 75	55.9 [25.7, 86.4]	7.2	51.2 [23.5, 79.1]	6.4	70.0 [32.2, 108.2]	8.4	44.5 [20.4, 68.7]	5.5	60.6 [27.8, 93.5]	8.1
≥ 60^a^	28.7	4.8	26.9	4.1	37.9	5.8	23.5	3.7	31.7	5.2

*Note:* Negative CI estimates were suppressed to zero because of biological implausibility.

Abbreviations: CI: confidence interval; IR: incidence rate extrapolated to the national level; NA: not applicable due to data not suitable for modeling.

^a^ Based on pooling results for models fitted to adults for included age groups; thus, confidence intervals are not provided.

**TABLE 2:** Estimated incidence rate per 100,000 person‐years of RSV‐attributable cardiovascular hospitalizations and percentage (%) of all respective cardiovascular hospitalizations attributable to RSV infections in adults in Germany, 2015–2019.

Age groups (years)	2015		2016		2017		2018		2019	
	IR [95% CI]	%	IR [95% CI]	%	IR [95% CI]	%	IR [95% CI]	%	IR [95% CI]	%
Chronic heart failure exacerbation										
18–44	NA		NA		NA		NA		NA	
45–59	NA		NA		NA		NA		NA	
60–74	65.9 [14.3, 117.0]	2.3	58.2 [12.6, 103.4]	1.9	87.1 [18.9, 154.6]	2.8	52.1 [11.3, 92.4]	1.8	71.4 [15.5, 126.7]	2.4
≥ 75	302.5 [76.6, 542.1]	1.9	300.7 [76.2, 538.8]	1.8	383.8 [97.2, 687.8]	2.3	250.0 [63.3, 448.1]	1.5	335.1 [84.9, 600.6]	2.1
≥ 60^a^	127.9	2.0	123.0	1.8	168.7	2.5	105.3	1.6	141.7	2.2
Ischemic heart diseases										
18–44	NA		NA		NA		NA		NA	
45–59	15.4 [0, 34.0]	1.6	15.2 [0, 33.5]	1.5	23.3 [0, 51.5]	2.2	15.0 [0, 33.1]	1.5	21.4 [0, 47.2]	2.1
60–74	92.1 [0.6, 175.4]	1.8	87.2 [0.6, 166.0]	1.6	124.4 [0.8, 236.8]	2.5	76.5 [0.5, 145.7]	1.6	103.1 [0.7, 196.3]	2.1
≥ 75	357.9 [47.9, 672.1]	1.9	327.7 [43.9, 615.5]	1.6	448.2 [60.0, 841.8]	2.3	284.5 [38.1, 534.4]	1.5	387.5 [51.9, 727.9]	2.1
≥ 60^a^	162.5	2.0	152.6	1.8	214.5	2.5	133.3	1.6	179.8	2.2
Arrhythmia										
18–44	NA		NA		NA		NA		NA	
45–59	16.3 [1.8, 31.8]	2.5	15.0 [1.7, 29.2]	2.0	24.2 [2.7, 47.2]	3.1	15.1 [1.7, 29.5]	2.0	22.0 [2.5, 42.8]	2.9
60–74	108.7 [26.5, 175.8]	2.6	96.1 [23.5, 155.4]	2.2	143.7 [35.1, 232.4]	3.3	85.9 [21.0, 138.9]	2.1	117.8 [28.7, 190.5]	2.8
≥ 75	460.9 [118.3, 786.7]	2.1	394.2 [101.1, 672.8]	1.6	565.2 [145.0, 964.6]	2.4	348.6 [89.5, 595.0]	1.5	483.1 [124.0, 824.5]	2.0
≥ 60^a^	201.5	2.3	176.6	1.8	260.4	2.7	157.2	1.7	215.8	2.3
Cerebrovascular diseases										
18–44	NA		NA		NA		NA		NA	
45–59	NA		NA		NA		NA		NA	
60–74	NA		NA		NA		NA		NA	
≥ 75	97.6 [9.9, 189.7]	1.7	83.5 [8.5, 162.2]	1.4	119.7 [12.1, 232.6]	2.0	73.9 [7.5, 143.5]	1.3	102.3 [10.4, 198.8]	1.9

*Note:* Negative CI estimates were suppressed to zero because of biological implausibility.

Abbreviations: CI: confidence interval; IR: incidence rate extrapolated to the national level; NA: not applicable due to data not suitable for modeling.

^a^ Based on pooling results for models fitted to adults for included age groups; thus, confidence intervals are not provided.

The estimated annual IR of RSV‐attributable respiratory hospitalizations in the age group ≥ 60 years was highest for chronic lower respiratory tract diseases (range across the study period: 103–168 hospitalizations per 100,000) and bronchitis/bronchiolitis (77–122 hospitalizations per 100,000) (Table 1). The annual IRs of RSV‐attributable bronchitis/bronchiolitis hospitalizations for those aged ≥ 75 years were approximately 11‐ to 12‐fold and 32‐ to 43‐fold higher than the rates for adults aged 60–74 and 45–59 years, respectively. Similarly, the rates of chronic lower respiratory diseases were approximately 3‐ to 4‐fold and 12‐ to 15‐fold higher in the oldest age group (≥ 75 years) compared to younger age groups (60–74 and 45–59 years) (Table 1).

The proportions of different clinical syndromes attributable to RSV were comparable between age groups and respiratory subgroups (range 1%–5% in age groups 45–59 years and 60–74 years and 3%–8% in ≥ 75 years) with the exception of bronchitis or bronchiolitis, where the RSV‐attributable proportion was 1%–10% for younger age groups and 17%–25% for adults ≥ 75 years (Table 1). For cardiovascular causes, arrhythmia (157–260 per 100,000 person‐years) and ischemic heart diseases (133–215 per 100,000 person‐years) followed by chronic heart failure (105–169 per 100,000 person‐years) showed the highest RSV‐attributable annual IRs (Table 2) in adults aged ≥ 60 years. The RSV‐attributable IRs were notably higher in older age groups, particularly among hospitalized individuals aged ≥ 75 years. The IRs of RSV‐attributable arrhythmia and ischemic heart disease hospitalizations for those aged ≥ 75 years were both approximately 4‐fold higher than the rates for adults aged 60–74 years. Moreover, these IRs were about 25‐fold and 21‐fold higher, respectively, than for adults aged 45–59 years (Table 2). RSV‐attributable proportions were comparable between age groups and cardiovascular subgroups, accounting for approximately 1% to 3% of all hospitalizations (Table 2).

## 4 Discussion

Our study complements the results of the first model‐based study in Germany [24] by providing estimates on specific types of cardiorespiratory hospitalizations attributable to RSV. We found a high burden of cardiovascular hospitalizations related to RSV, with RSV‐attributable IRs in adults aged ≥ 60 years for arrhythmia (157–260 per 100,000 person‐years) and ischemic heart diseases (133–215 per 100,000 person‐years). Among respiratory conditions, chronic lower respiratory tract diseases (103–168 per 100,000 person‐years) and bronchitis/bronchiolitis (77–122 per 100,000 person‐years) had the highest RSV‐attributable incidences. As in other studies, we found that RSV‐attributable IRs increased with age, with a steeper inflection upwards observed in patients aged ≥ 60 years. Our estimates were much higher than those based on RSV‐specific ICD‐10 codes alone, supporting other evidence that ICD‐based studies substantially underestimate the RSV burden in adults [9, 22]. Lower RSV hospitalization IRs in adults aged > 59 years (0.11–11.36 per 100,000 person‐years) were also reported in an observational study in Germany, which was based on data derived from the primary discharge diagnosis of RSV (J21.1, J20.5, J21.0) [35].

Our study adds to the growing evidence of the association between RSV circulation and cardiovascular events. We found that approximately 2% to 3% of hospitalizations due to arrhythmia, ischemic heart diseases, and chronic heart failure in people aged ≥ 60 years were attributable to RSV. In the oldest age group (≥ 75 years), we also found an association—in line with another study [22]—between RSV and cerebrovascular diseases, with 1%–2% of those hospitalizations being attributable to RSV. For instance, in Spain, the RSV‐attributable proportion of ischemic heart diseases in adults aged ≥ 60 years (3%) and cerebrovascular diseases in adults aged ≥ 80 years (1%) was comparable to our results [22]. Although the RSV‐attributable proportions were comparable, we report higher IRs in our study due to higher observed hospitalizations for all cardiovascular causes in Germany. This in turn might be related to different coding practices or different prevalences of comorbidities in those two populations.

Several mechanisms may explain the relationship between RSV and cardiovascular events. Part of those conditions may be related to the indirect effect of inflammation due to acute respiratory infections. Inflammation may increase the concentration of C‐reactive protein, inflammatory cytokines, and clotting factors such as fibrinogen, leading to an increased risk of thrombotic coronary occlusion [16, 36, 37]. Additionally, RSV may cause endothelial dysfunction and inhibit the function of vasodilating nitric oxide or prostaglandins, contributing to arterial and venous thromboembolic disease [38]. Development of pulmonary hypertension may lead to secondary myocardial dysfunction, hypotension, and arrhythmia [20]. RSV can also directly infect organs; for example, it was isolated from myocardial tissue biopsies in patients with myocarditis [15, 20]. Cardiovascular diseases remain the leading cause of morbidity and mortality worldwide [39]. Thus, the potential impact of a vaccine preventing cardiovascular events, as observed for the influenza vaccine [40, 41], might be substantial.

Our estimates for RSV‐attributable IRs hospitalizations due to bronchitis/bronchiolitis (77–122 per 100,000) and influenza/pneumonia diagnosis grouping (67–98 per 100,000) in adults aged ≥ 60 years align with the study conducted in Spain (100–110 per 100,000 and 83–91, respectively) [22]. The Spanish study utilized the same generic protocol that we used for this study [33]. In contrast, when compared to another model‐based study from the United Kingdom, IRs in older age groups (65–74 and ≥ 75 years) were considerably higher in Germany [6]. Our study differed from the UK study in that we used quasi‐Poisson regression modeling weekly counts versus linear regression modeling weekly outcome rates in the United Kingdom; additionally, we used an RSV proxy definition of weekly hospitalizations due to RSV, and the UK study used weekly RSV counts from the surveillance system. We used RSV hospitalization data as a proxy because hospitalization data are less affected by testing practices than surveillance data, which will be mostly driven by influenza (data not shown). Further, the UK study used only primary diagnosis codes, which have been shown to underestimate incidence in validation studies [42].

Among age groups 45–59 and 60–74 years, the highest RSV‐attributable IR was identified for chronic lower respiratory tract diseases. Respiratory viruses, including RSV, are known causes of exacerbation of COPD [12]. In prospective cohort studies, approximately 4%–11% of COPD patients tested positive for RSV [43, 44]. The prevalence of COPD is increasing globally, leading to enormous healthcare costs and high mortality [45, 46]. Exacerbation of COPD is associated with an accelerated decline in lung function, progression of the disease, and lower quality of life [11]. Thus, preventing COPD exacerbation should be a public health priority, and vaccination of COPD patients should be prioritized, as suggested by the recent recommendation from the US Centers for Disease Control and Prevention [47].

The main strength of our study was the use of a large database of approximately 3.5 million insured persons, an extended study period of 5 years, and the inclusion of eight different medical conditions, allowing for a more sensitive estimation of the RSV burden. In addition, our study was based on a generic protocol [33] that permits robust comparisons between countries. The protocol was developed based on an extensive prior literature review and experts' input.

We also acknowledge some study limitations. Our model included only RSV and influenza as viral indicators, assuming those are the only pathogens related to the outcome of interest. However, by including the periodic component and overdispersion parameter, we indirectly accounted for other potentially relevant pathogens. Second, RSV hospitalizations in children less than 2 years old as the RSV indicator may not fully reflect viral activity in adults. However, the community RSV activity pattern in adults is similar to that observed in children, and for the model performance, the most important factor is the fluctuation over time rather than the absolute numbers. Third, although the SHI database is representative of the German population, the age distribution is slightly skewed towards young individuals. To account for this difference, we used correction factors to standardize for age in the incidence calculation. Lastly, we did not limit the population denominators to those individuals at risk of a specific condition (e.g., limit the denominator for the RSV‐attributable COPD events to those with COPD diagnosis), which would have increased the reported incidence rate. Lastly, as this is based on modeling RSV disease burden from a series of community‐level indicators, we cannot prove the causality.

Our study showed a high and largely unrecognized cardiovascular and respiratory burden of RSV among adults in Germany. Substantial underestimation of RSV cases from the use of ICD coding and administrative databases emphasizes the need for standard‐of‐care testing among adults with LRTI symptoms, exacerbations of chronic lung disease, and cardiovascular symptoms. Newly introduced vaccines to prevent lower respiratory tract disease caused by RSV in adults ≥ 60 years might have a high impact, not only on respiratory hospitalizations but also on cardiac hospitalizations. Two RSV vaccines (GSK's RSV Arexvy vaccine [48] and Pfizer's RSV Abrysvo vaccine [49]) have been available in Germany since 2023. In 2024, a third RSV vaccine (Moderna's mRESVIA vaccine) received market authorization [50].

## Author Contributions

**Caihua Liang:** data curation, methodology, project administration, validation, supervision, writing – review and editing. **Aleksandra Polkowska‐Kramek:** methodology, data curation, project administration, writing – review and editing, writing – original draft. **Caroline Lade:** methodology, data curation, project administration, writing – review and editing. **Lea J. Bayer:** methodology, data curation, project administration, writing – review and editing. **Robin Bruyndonckx:** data curation, formal analysis, methodology, software, writing – original draft, writing – review and editing. **Bennet Huebbe:** methodology, project administration, writing – review and editing. **Worku Biyadgie Ewnetu:** methodology, data curation, formal analysis, software, visualization, writing – review and editing. **Pimnara Peerawaranun:** methodology, formal analysis, software, visualization, writing – review and editing, validation. **Maribel Casas:** methodology, writing – original draft, writing – review and editing. **Thao Mai Phuong Tran:** methodology, data curation, formal analysis, software, writing – review and editing. **Gordon Brestrich:** methodology, writing – review and editing. **Christof von Eiff:** methodology, writing – review and editing. **Bradford D. Gessner:** conceptualization, methodology, writing – review and editing. **Elizabeth Begier:** conceptualization, methodology, writing – review and editing, supervision. **Gernot Rohde:** conceptualization, methodology, writing – review and editing.

## Ethics Statement

This study used aggregated and anonymized data; therefore, it did not require approval from institutional review boards or ethical committees or informed consent from patients. The study was conducted following legal and regulatory requirements and research practices described in the Good Epidemiological Practice guidelines issued by the International Epidemiological Association.

## Conflicts of Interest

Aleksandra Polkowska‐Kramek, Robin Bruyndonckx, Worku Biyadgie Ewnetu, Pimnara Peerawaranun, Maribel Casas, and Thao Mai Phuong Tran are employees of P95 Epidemiology & Pharmacovigilance, which received funding from Pfizer in connection with the development of this manuscript and to conduct the research described in this manuscript. Caihua Liang, Caroline Lade, Lea J. Bayer, Bennet Huebbe, Gordon Brestrich, Christof von Eiff, Bradford D. Gessner, and Elizabeth Begier are Pfizer employees and may own Pfizer stock. Gernot Rohde is an expert in the field of RSV and received an honorarium from Pfizer for input on the study design.

## Supporting information


**Table S1.** Number of hospitalized cases stratified by age group in Germany, 2015–2019.

## Data Availability

The datasets generated and/or analyzed during this study are not publicly available (data provider's restrictions). Weekly counts of hospitalized cases and extrapolation factors were provided by Gesundheitsforen Leipzig GmbH.
