# Supplementary material for: Estimated Incidence Rate of Specific Types of Cardiovascular and Respiratory Hospitalizations Attributable to Respiratory Syncytial Virus Among Adults in Germany Between 2015 and 2019
Source: Influenza Other Respir Viruses. 2025 Apr 28;19(5):e70097. doi: 10.1111/irv.70097 (PMC12035416; doi:10.1111/irv.70097)
Supplement: Supplementary file 1 — Table S1. Number of hospitalized cases stratified by age group in Germany, 2015–2019. [file IRV-19-e70097-s001.docx]

**Supplementary Material**

**Title: Estimated incidence rate of specific types of cardiovascular and respiratory hospitalizations attributable to Respiratory Syncytial Virus among adults in Germany between 2015 and 2019**

**Author Details:**

Caihua Liang^1^, Aleksandra Polkowska-Kramek^2^, Caroline Lade^3^, Lea J. Bayer^3^, Robin Bruyndonckx^2^, Bennet Huebbe^3^, Worku Biyadgie Ewnetu^2^, Pimnara Peerawaranun^2^, Maribel Casas^2^, Thao Mai Phuong Tran^2^, Gordon Brestrich^3^, Christof von Eiff^3^, Bradford D. Gessner^1^, Elizabeth Begier^4^, Gernot Rohde^5^

**Affiliations:**

^1^ Pfizer Inc-New York (USA)

^2^ P95 Epidemiology & Pharmacovigilance, Leuven, Belgium

^3^ Pfizer Pharma GmbH - Berlin (Germany)

^4^ Pfizer Inc - Dublin (Ireland)

^5^ Goethe University Frankfurt, University Hospital, Medical Clinic I, Department of Respiratory Medicine, Frankfurt/Main, Germany

**Corresponding author:**

Caihua Liang

Email: [caihua.liang@pfizer.com](mailto:caihua.liang@pfizer.com)

Phone: 1-716-429-5598

Address: 66 Hudson Blvd E, New York, NY 10001

**Supplementary Table 1. Number of hospitalized cases stratified by age group in Germany, 2015-2019**

|  | Age group | | | |  |
| --- | --- | --- | --- | --- | --- |
| Outcome | 18-44 years | 45-59 years | 60-74 years | ≥75 years | Total |
| Influenza/pneumonia | | | | |  |
| 2015^§^ | 848 | 1893 | 3557 | 5745 | 12043 |
| 2016 | 1043 | 2149 | 4035 | 6527 | 13754 |
| 2017 | 957 | 2258 | 4550 | 7733 | 15498 |
| 2018 | 1090 | 2498 | 4860 | 8112 | 16560 |
| 2019 | 959 | 2277 | 4948 | 8025 | 16209 |
| Total | 4897 | 11075 | 21950 | 36142 | 74064 |
| Bronchitis/bronchiolitis | | | | |  |
| 2015^§^ | 360 | 561 | 925 | 1499 | 3345 |
| 2016 | 374 | 631 | 947 | 1584 | 3536 |
| 2017 | 358 | 706 | 1116 | 2105 | 4285 |
| 2018 | 414 | 737 | 1233 | 1995 | 4379 |
| 2019 | 346 | 707 | 1162 | 1875 | 4090 |
| Total | 1852 | 3342 | 5383 | 9058 | 19635 |
| Chronic lower respiratory tract diseases | | | | |  |
| 2015^§^ | 2319 | 5918 | 10069 | 8722 | 27028 |
| 2016 | 2506 | 6529 | 11440 | 10380 | 30855 |
| 2017 | 2613 | 6936 | 12608 | 11626 | 33783 |
| 2018 | 2668 | 7054 | 13517 | 11630 | 34869 |
| 2019 | 2572 | 7130 | 14102 | 12005 | 35809 |
| Total | 12678 | 33567 | 61736 | 54363 | 162344 |
| Upper respiratory tract diseases | | | | |  |
| 2015^§^ | 5453 | 3228 | 1668 | 804 | 11153 |
| 2016 | 5892 | 3589 | 1952 | 911 | 12344 |
| 2017 | 5974 | 3780 | 2154 | 1086 | 12994 |
| 2018 | 6107 | 3853 | 2315 | 1141 | 13416 |
| 2019 | 5907 | 3819 | 2347 | 1098 | 13171 |
| Total | 29333 | 18269 | 10436 | 5040 | 63078 |
| Chronic heart failure exacerbation | | | | |  |
| 2015^§^ | 592 | 3744 | 9333 | 16411 | 30080 |
| 2016 | 673 | 4094 | 10595 | 19283 | 34645 |
| 2017 | 728 | 4483 | 11653 | 21816 | 38680 |
| 2018 | 710 | 4603 | 12175 | 22957 | 40445 |
| 2019 | 715 | 4550 | 13027 | 23777 | 42069 |
| Total | 3418 | 21474 | 56783 | 104244 | 185919 |
| Ischemic heart diseases | | | | |  |
| 2015^§^ | 694 | 7104 | 16212 | 19442 | 43452 |
| 2016 | 722 | 7846 | 18213 | 22615 | 49396 |
| 2017 | 729 | 8161 | 19209 | 25073 | 53172 |
| 2018 | 768 | 8446 | 20014 | 26228 | 55456 |
| 2019 | 800 | 8466 | 21432 | 26878 | 57576 |
| Total | 3713 | 40023 | 95080 | 120236 | 259052 |
| Arrhythmia | | | | |  |
| 2015^§^ | 1370 | 4826 | 13433 | 22842 | 42471 |
| 2016 | 1522 | 5741 | 15257 | 27226 | 49746 |
| 2017 | 1575 | 5953 | 16563 | 31096 | 55187 |
| 2018 | 1642 | 6231 | 17298 | 32930 | 58101 |
| 2019 | 1595 | 6239 | 18472 | 34652 | 60958 |
| Total | 7704 | 28990 | 81023 | 148746 | 266463 |
| Cerebrovascular diseases | | | | |  |
| 2015^§^ | 427 | 1870 | 3974 | 6066 | 12337 |
| 2016 | 423 | 2086 | 4380 | 6949 | 13838 |
| 2017 | 462 | 2164 | 4537 | 7760 | 14923 |
| 2018 | 479 | 2291 | 4849 | 8024 | 15643 |
| 2019 | 462 | 2166 | 5042 | 8013 | 15683 |
| Total | 2253 | 10577 | 22782 | 36812 | 72424 |

^§^ Weeks 1-5 are not included in the analysis as insufficient information on the RSV proxy is available for these first weeks.
